# Comparison of Survival Outcomes of Single- and Five-Fraction Schedules of Stereotactic Body Radiation Therapy for Early-Stage Central or Peripheral NSCLC

**DOI:** 10.3390/cancers15061648

**Published:** 2023-03-08

**Authors:** Karen Huang, Sharan Prasad, Sung Jun Ma, Austin J. Iovoli, Mark K. Farrugia, Nadia K. Malik, Anurag K. Singh

**Affiliations:** 1 Department of Radiation Medicine, Roswell Park Comprehensive Cancer Center, Elm and Carlton Streets, Buffalo, NY 14263, USA; 2 College of Human Ecology, Cornell University, 410 Thurston Avenue, Ithaca, NY 14850, USA

**Keywords:** NSCLC, SBRT, central, peripheral, survival, single fraction

## Abstract

**Simple Summary:**

Patients with early-stage non-small cell lung cancer (NSCLC) treated with stereotactic body radiation therapy (SBRT) often receive different treatment regimens based on the location of the tumor to minimize the risk of severe side effects. The aim of our study was to examine survival outcomes of patients treated with single-fraction SBRT for peripheral tumors and five-fraction SBRT for central tumors. In a cohort of 265 patients with NSCLC, we found no differences in patients treated for peripheral versus central tumors in progression-free survival, overall survival, local failure, nodal failure, or distant failure. These findings were confirmed upon propensity score matching. Our study demonstrated survival outcomes of patients treated with SBRT for early-stage NSCLC were equivalent for central and peripheral tumors.

**Abstract:**

Background: The treatment of early-stage non-small cell lung cancer (NSCLC) with stereotactic body radiation therapy (SBRT) frequently involves different fractionation schemes for peripheral and central tumors due to concerns with toxicity. We performed an observational cohort study to determine survival outcomes for patients with peripheral and central NSCLC treated with SBRT. Methods: A single-institutional database of patients with early-stage NSCLC treated with SBRT from September 2008 to December 2018 was evaluated. Outcomes were progression-free survival (PFS), overall survival (OS), local failure (LF), nodal failure (NF), and distant failure (DF). Cox multivariable analysis (MVA), Kaplan–Meier plotting, Fine–Gray competing risk MVA, and propensity score matching were performed. Results: A total of 265 patients were included with a median follow-up of 44.2 months. There were 191 (72%) and 74 (28%) patients with peripheral and central tumors treated with single-fraction SBRT to a dose of 27 Gy and five-fraction SBRT to a dose of 50 Gy, respectively. On Cox MVA, there was no difference in OS (adjusted hazards ratio (aHR) of 1.04, 95% CI of 0.74–1.46) or PFS (aHR of 1.05, 95% CI of 0.76–1.45). On Fine–Gray competing risk MVA, there were no differences in LF, NF, or DF. Propensity matching confirmed these findings. Conclusion: The survival outcomes of patients treated with SBRT for early-stage NSCLC were equivalent for central and peripheral tumors.

## 1. Introduction

The leading cause of cancer-related deaths worldwide is lung cancer, accounting for 18% of cancer-related deaths in 2020 [1]. For early-stage non-small cell lung cancer (NSCLC), stereotactic body radiation therapy (SBRT) remains the standard of care in medically inoperable patients, with five-year survival rates ranging from 20–40% [2,3,4]. Various SBRT regimens ranging from 1 to 5 fractions are commonly used for early-stage NSCLC [2,5,6,7].

Due to proximity to critical structures such as the proximal bronchial airway, heart, and esophagus, SBRT for central tumors is currently thought to increase the risk of normal tissue toxicity. In 2006, significant excessive toxicity was reported for early-stage, central NSCLC lung tumors treated with three-fraction SBRT [8]. Further analysis of this trial showed that the excess toxicity associated with central tumors was no longer statistically significant and that survival was not changed by peripheral versus central location [9]. Multiple subsequent studies have identified central lung tumors to be associated with high rates of toxicity [10,11].

Despite the updated findings of the Timmerman trial, concerns about toxicity with central lung SBRT persist. Since the initial finding, later studies of SBRT have made the central lung a “no-fly zone” and focused on either peripheral or central lung tumors. Consequently, little is known about the long-term outcomes of central versus peripheral tumors treated with SBRT. To compare central versus peripheral NSCLC SBRT on survival and recurrence outcomes, we performed a single-institution, observational cohort study. 

## 2. Methods

The cohort study was approved by the Roswell Park Comprehensive Cancer Center Institutional Review Board (EDR 171710). The Strengthening the Reporting of Observations Studies in Epidemiology (STROBE) reporting guideline was adhered to.

The patient database was selected from patients diagnosed with early-stage NSCLC (T1-2N0M0) who underwent 27 Gy in 1 fraction for peripheral tumors or 50 Gy/5 fractions for central tumors with heterogeneity correction at Roswell Park Comprehensive Cancer Center between September 2008 and December 2018. Tumor centrality was defined per RTOG 0813 as tumors within or touching the zone 2 cm around the proximal bronchial tree (PBT) or immediately adjacent to the mediastinal or pericardial pleura [11]. Candidacy for surgical resection was determined at the discretion of the thoracic surgeon.

All patients underwent SBRT as previously described [5]. Patients underwent CT simulation in the supine position with arms above their head using a thoracic Medical Intelligence BodyFIX^®^ immobilization system (Elekta, Stockholm, Sweden). Tumor motion management included either abdominal compression or respiratory gating, as previously described [12,13]. Dose delivery techniques employed included non-coplanar 3-dimensional conformal fields (3DCRT) or volumetric modulated arc therapy (VMAT). Heterogeneity corrections were used only for patients treated with intensity-modulated radiation therapy. Normal tissue dose constraints from RTOG 0915 were utilized for patients treated with 1 fraction and constraints from RTOG 0813 were used for patients treated with 5 fractions [2,11].

Clinically relevant variables, including age, gender, race, Karnofsky Performance Status (KPS), histology (adenocarcinoma, squamous cell carcinoma, NSCLC not otherwise specified), T stage, smoking status, operability, and tumor location were obtained retrospectively. Race was categorized as White, African American, Asian, American Indian/Alaska Native, unknown, or declined to answer. Toxicity data were unavailable. Due to the small sample size, non-White patients were grouped as a single category. There were no missing values in our database in such variables when analyses were performed.

Overall survival (OS) and progression-free survival (PFS) were the primary outcomes. OS was determined from time between the start of SBRT to the last known follow-up or death from any cause. PFS was determined from the time between the start of SBRT to any tumor recurrence or, if none, the last known follow-up or death. Secondary outcomes were local failure (LF), nodal failure (NF), and distant failure (DF). LF was defined per RTOG 0813 as local enlargement confirmed by positron emission tomography (PET) scan or biopsy, marginal failure, or involved lobe failure [11]. NF was defined as tumor recurrence in any thoracic nodal station and DF as any extra-thoracic or contralateral lung recurrence. All recurrences were evaluated in a multidisciplinary setting based on radiographic findings and, if available, biopsy results of metastatic sites.

### Statistical Analysis

A Fisher exact test and Mann–Whitney U test were performed to compare baseline characteristics among the cohorts with different fractions. All categorical variables were handled as unordered. To evaluate OS and PFS, Cox multivariable analysis (MVA), Kaplan–Meier plotting, and a log-rank test were performed. Fine–Gray competing risk MVA was used to evaluate LF, NF, and DF with death as a competing event. All MVA models included previously listed clinically relevant variables.

To reduce selection bias, propensity score matching was performed between central and peripheral tumor cohorts. This technique attempts to balance treatment group confounding factors to make them comparable so conclusions can be drawn about the casual impact of a treatment on outcomes using observational data. Cohorts were matched based on the variables previously listed above. Matching was based on the nearest-neighbor method in a 1:1 ratio without replacement with a caliper distance of 0.2 [14]. After matching, Kaplan–Meier plot, Cox, and Fine–Gray regression models were performed to evaluate OS, PFS, LF, NF, and DF. All statistical tests were two-sided and *p* < 0.05 was considered statistically significant. Data analyses were conducted using R (version 4.1.2, R Project for Statistical Computing, Vienna, Austria).

## 3. Results

A total of 265 patients (142 female (53.6%); median (interquartile range) age of 77 (70–82) years met our criteria (Table 1). There were 74 (27.9%) and 191 (72.1%) patients with central tumors treated with five-fraction SBRT and peripheral tumors treated with single-fraction SBRT, respectively. The median follow-up was 44.2 months (interquartile range (IQR) of 26.7–61.2). Baseline characteristics were well balanced among these cohorts, except that peripheral tumors trended towards having fewer T2 stages. Most patients had adenocarcinoma (139, 52.5%) and squamous cell carcinoma (106, 40.0%). The majority of the tumors were T1 (211, 79.6%). 

**Figure 1:**
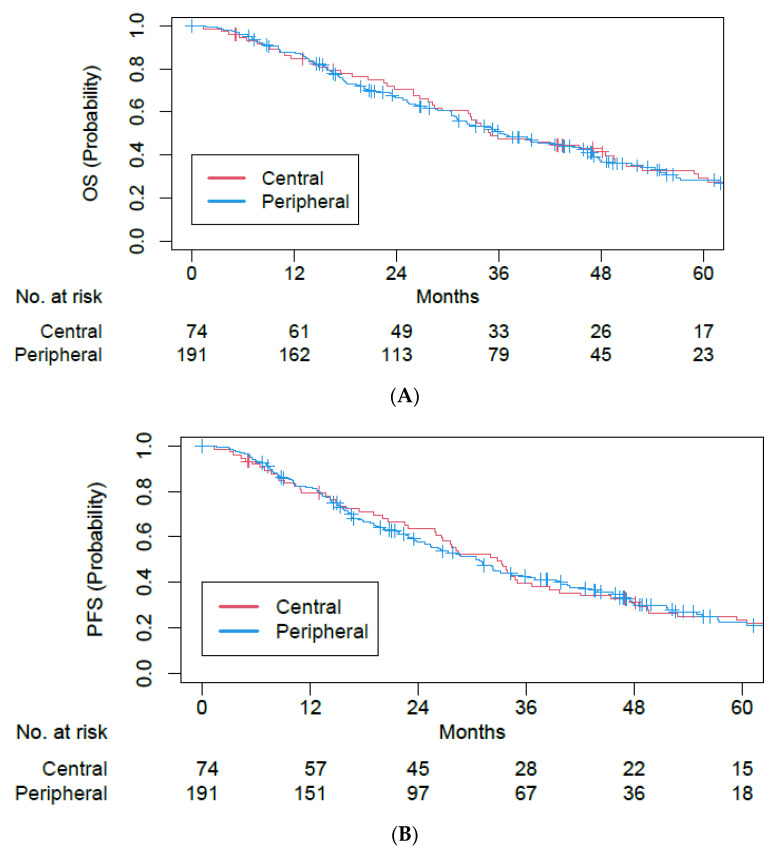
Kaplan–Meier plots for (**A**) overall survival and (**B**) progression-free survival among single- and five-fraction SBRT cohorts.

On Cox MVA of the entire cohort of patients with central and peripheral tumors (Table 2), there was no statistically significant difference in OS (peripheral vs. central tumors: adjusted hazards ratio (aHR) of 1.04, 95% confidence interval (CI) of 0.74–1.46, *p* = 0.81) and PFS (peripheral vs. central tumors: aHR of 1.05, 95% CI of 0.76–1.45, *p* = 0.77). 5-year OS rates were 29.3% and 28.3%, while 5-year PFS rates were 23.3% and 22.3% for central and peripheral tumors, respectively (Figure 1). Female gender, good KPS, and former smoking status compared to current smoking status were associated with improved OS and PFS (Table 2). 

**Figure 2:**
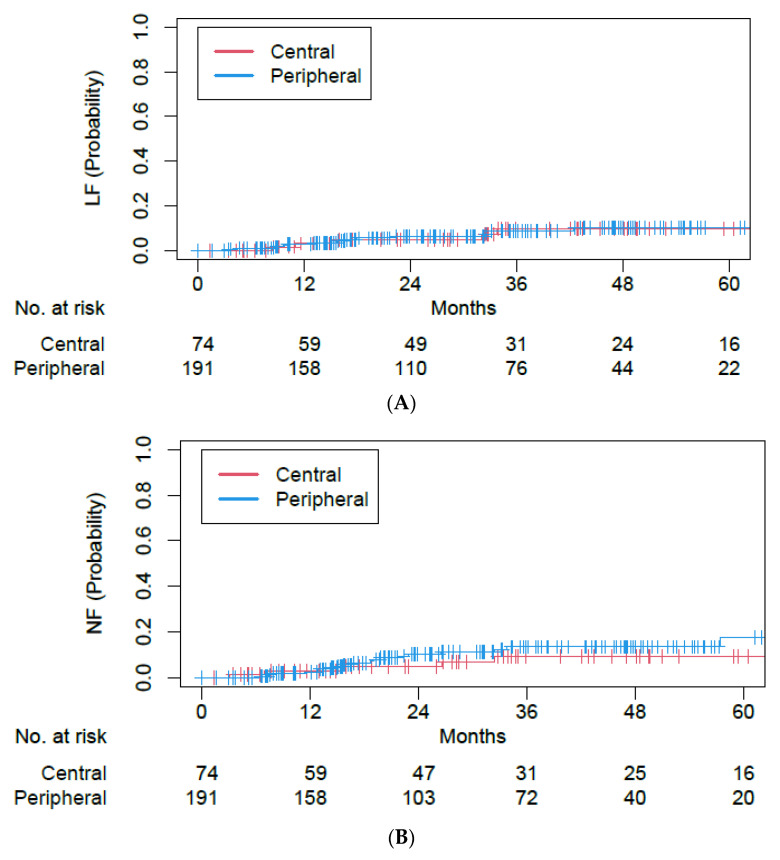

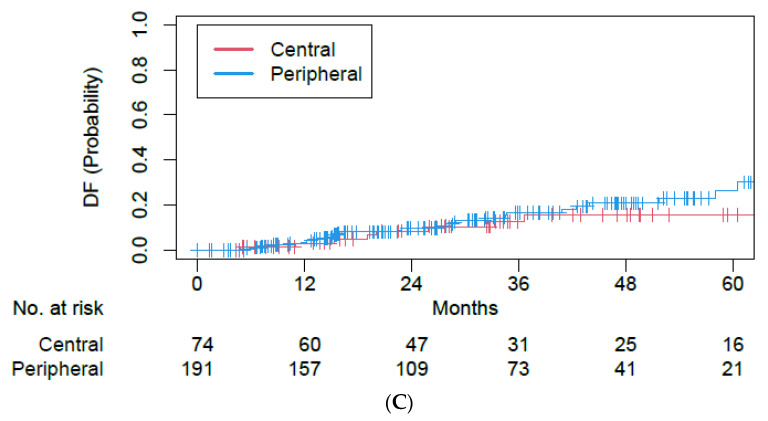
Cumulative incidence plots for (**A**) local failure, (**B**) nodal failure, and (**C**) distant failure outcomes among single- and five-fraction SBRT cohorts.

On Fine–Gray competing risk MVA (Table 3), similar findings were seen for LF (peripheral vs. central tumors: aHR of 1.03, 95% CI of 0.37–2.83, *p* = 0.95), NF (peripheral vs. central tumors: aHR of 1.77, 95% CI of 0.63–4.94, *p* = 0.28), and DF (peripheral vs. central tumors: aHR of 1.60, 95% CI of 0.76–3.36, *p* = 0.22). 5-year LF, NF, and DF rates were 9.7%, 9.1%, and 15.5% for central tumors; they were 10.1%, 17.5%, and 26.6% for peripheral tumors (Figure 2). NSCLC with histology not otherwise specified and never smokers were associated with less LF and NF (Table 3), in part due to a lack of events in small subgroup sample sizes of 20 and 12 patients, respectively. 

**Figure 3:**
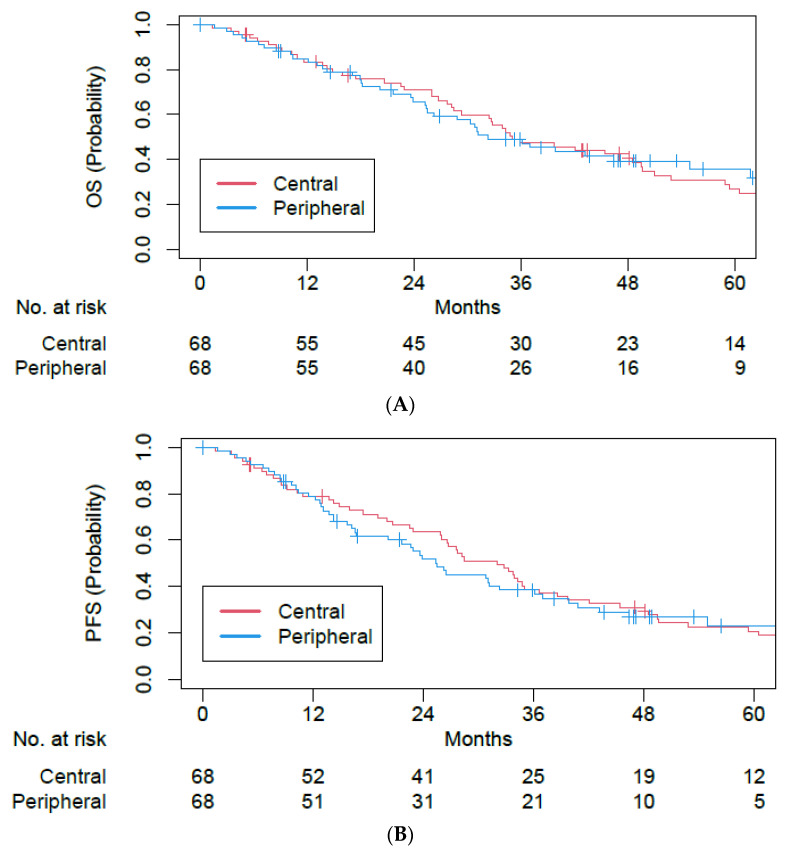
Kaplan–Meier plots for (**A**) overall survival and (**B**) progression-free survival among single- and five-fraction SBRT cohorts after propensity score matching.

**Figure 4:**
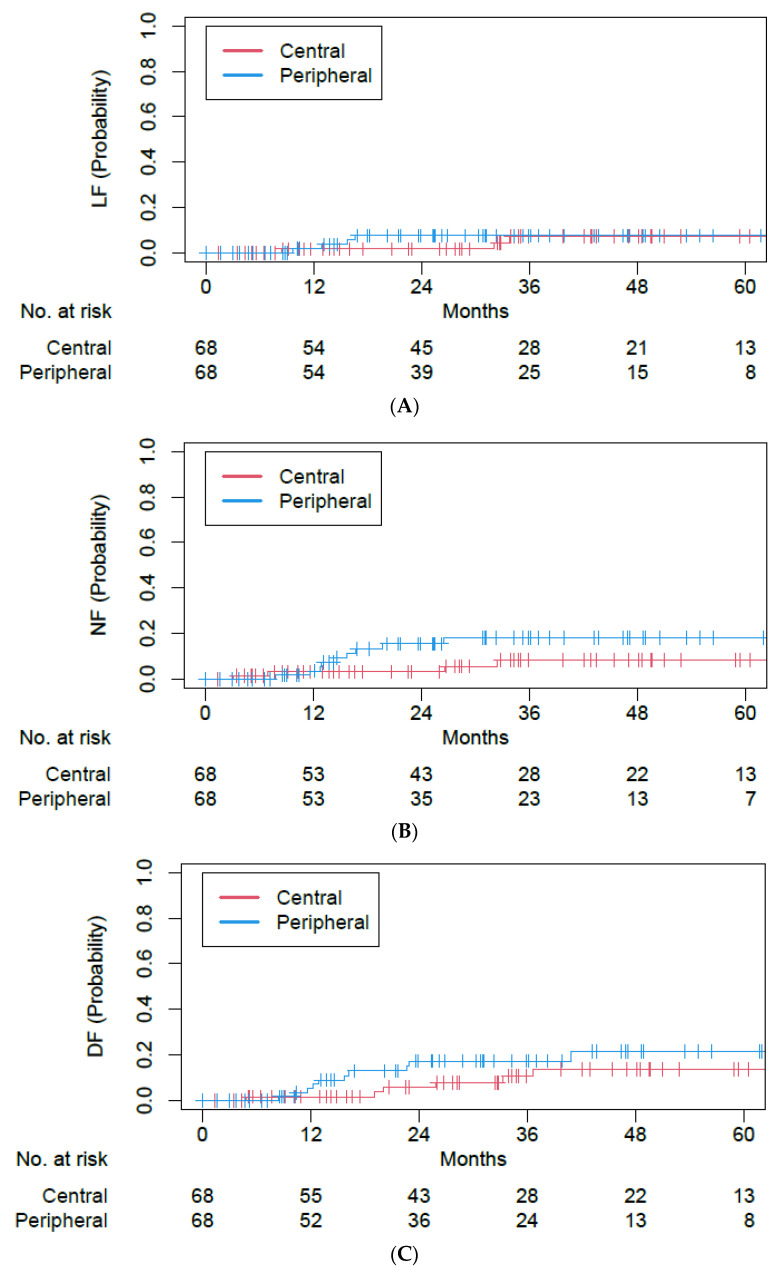
Cumulative incidence plots for (**A**) local failure, (**B**) nodal failure, and (**C**) distant failure outcomes among single- and five-fraction SBRT cohorts after propensity score matching.

After propensity score matching, 68 matched pairs were identified (Table 4). The median follow-up was 47.1 months (IQR 30.6–63.4). Among central versus peripheral tumor matched cohorts, there was no statistically significant difference in OS (5-year OS 27.0% vs. 35.7%; hazards ratio (HR) of 1.07, 95% CI of 0.71–1.62, *p* = 0.75; Figure 3), PFS (5-year PFS 20.7% vs. 23.0%; HR of 1.13, 95% CI of 0.77–1.67, *p* = 0.53; Figure 3), LF (5-year LF 7.1% vs. 7.5%; HR of 1.43, 95% CI of 0.34–6.07, *p* = 0.62; Figure 4), NF (5-year NF 8.2% vs. 18.1%; HR of 2.49, 95% CI of 0.78–7.92, *p* = 0.12; Figure 4), or DF (5-year DF 13.7% vs. 21.4%; HR of 1.68, 95% CI of 0.66–4.28, *p* = 0.28; Figure 4).

## 4. Discussion

This study is the largest to report outcomes of central versus peripheral early-stage NSCLC cohorts treated with SBRT. We found no significant difference in OS or PFS between peripheral versus central tumors. When looking at secondary outcomes, we also observed no differences in LF, NF, or DF.

Two prospective randomized trials demonstrated single-fraction SBRT had the same local control, OS, and PFS outcomes as three- and four-fraction SBRT in peripheral early-stage NSCLC patients [2,12]. Retrospective analysis has shown that a single-fraction regimen is equivalent to five-fraction regimens for early-stage peripheral NSCLC [6]. Based on RTOG 0813, central early-stage NSCLC was treated with at least five fractions of SBRT at our institution [3,11].

Unfortunately, toxicity profiles among patients with central NSCLC tumors who underwent five-fraction SBRT were unavailable. High-grade toxicities have been reported among patients with such tumors [10,15,16]. In our study, central tumor SBRT outcomes were comparable to peripheral NSCLC tumors, consistent with prior retrospective studies [17,18]. These discrepancies may suggest a heterogeneous population of patients with central NSCLC tumors.

A further distinction exists for ultracentral early-stage NSCLC tumors, where the planning target volume (PTV) is abutting the proximal bronchial tree or touching mediastinal structures, such as the esophagus or great vessels. The Nordic HILUS trial reported a 18% grade 5 toxicity rate treating tumors ≤ 1 cm from the mainstem bronchus with SBRT [10]. A systematic review of SBRT for ultracentral lung lesions found treatment to be associated high local control rates but the potential for severe toxicity in patients receiving high doses to the proximal bronchial tree, those with endobronchial disease, and those receiving bevacizumab or anticoagulants around the time of SBRT [19]. In a matched cohort comparison between central and ultracentral lung tumors, a report found ultracentral tumor location correlated with reduced non-cancer-associated survival [20]. This could be due to increased cardiac toxicity from SBRT for ultracentral tumors, as another analysis of the same patient cohort found increased dose to the right atria to be associated with non-cancer-associated survival [21]. Despite these risks, another retrospective study found no difference in survival outcomes or toxicity between patients with central or ultracentral lung tumors [22].

### Limitations

Our study has multiple limitations due to its retrospective nature. Without toxicity data for the cohort, it is unclear whether patients with central tumors had differences in treatment toxicity that contributed to survival outcomes. Additionally, the reasons for undergoing SBRT, such as significant medical comorbidities or refusing surgery, were unavailable for analysis. Although the performance status variable was well balanced among different fractionation schemes even prior to propensity score matching, it is notable that patients treated with single-fraction SBRT had a higher number of patients with a KPS < 80.

## 5. Conclusions

Survival and tumor recurrence outcomes of patients treated with SBRT for early-stage NSCLC were equivalent for central tumors and peripheral tumors.

## Figures and Tables

**Table 1:** Baseline characteristics prior to propensity score matching.

	Central (*n* = 74)		Peripheral (*n* = 191)		
	N	%	N	%	*p*
Age					0.53
<65	11	14.9	22	11.5	
≥65	63	85.2	169	88.5	
Gender					0.22
Male	39	52.7	84	44.0	
Female	35	47.3	107	56.0	
Race					0.49
Caucasian	65	87.8	174	91.1	
Other	9	12.2	17	8.9	
KPS					0.08
<80	19	25.7	72	37.7	
≥80	55	74.3	119	62.3	
T stage					0.06
T1	53	71.6	158	82.7	
T2	21	28.4	33	17.3	
Histology					0.56
Adenocarcinoma	35	47.3	104	54.5	
Squamous cell carcinoma	33	44.6	73	38.2	
NSCLC (NOS)	6	8.1	14	7.3	
Smoking					0.69
Current	16	21.6	45	23.6	
Former	56	75.7	136	71.2	
Never	2	2.7	10	5.2	
Tumor location					0.56
LLL	11	14.9	31	16.2	
LUL	28	37.8	53	27.7	
RLL	14	18.9	36	18.8	
RML	2	2.7	9	4.7	
RUL	19	25.7	62	32.5	
Surgical Candidacy					0.44
Medically inoperable	51	68.9	121	63.4	
Poor pulmonary function	15	20.3	37	19.4	
Refused surgery	8	10.8	33	17.3	

NSCLC: non-small cell lung cancer; NOS: not otherwise specified; LLL: left lower lobe; LUL: left upper lobe; RLL: right lower lobe; RML: right middle lobe; RUL: right upper lobe.

**Table 2:** Multivariable Cox regression for overall and progression-free survival of the entire patient cohort (*n* = 265).

	Overall Survival			Progression-Free Survival		
	aHR	95% CI	*p*	aHR	95% CI	*p*
Location						
Central	Reference			Reference		
Peripheral	1.04	0.74–1.46	0.81	1.05	0.76–1.45	0.77
Age						
<65	Reference			Reference		
≥65	1.37	0.81–2.31	0.24	1.21	0.76–1.94	0.42
Gender						
Male	Reference			Reference		
Female	0.60	0.44–0.82	<0.01	0.62	0.46–0.84	<0.01
Race						
Caucasian	Reference			Reference		
Other	0.83	0.46–1.48	0.52	1.00	0.58–1.74	0.99
KPS						
<80	Reference			Reference		
≥80	0.53	0.38–0.74	<0.01	0.71	0.52–0.98	0.04
T stage						
T1	Reference			Reference		
T2	1.30	0.88–1.93	0.19	1.34	0.92–1.97	0.13
Histology						
Adenocarcinoma	Reference			Reference		
Squamous cell carcinoma	0.94	0.68–1.30	0.71	1.00	0.73–1.36	0.98
NSCLC (NOS)	1.01	0.58–1.75	0.98	1.10	0.64–1.87	0.73
Smoking						
Current	Reference			Reference		
Former	0.68	0.46–1.00	0.05	0.68	0.47–0.97	0.03
Never	0.66	0.29–1.53	0.33	0.60	0.26–1.35	0.21
Tumor location						
LLL	Reference			Reference		
LUL	0.80	0.50–1.29	0.36	0.77	0.48–1.23	0.28
RLL	0.83	0.50–1.37	0.47	0.86	0.52–1.40	0.54
RML	1.15	0.49–2.69	0.75	1.09	0.47–2.54	0.83
RUL	1.00	0.62–1.62	0.98	1.03	0.65–1.63	0.91

aHR: adjusted hazards ratio; CI: confidence interval; NSCLC: non-small cell lung cancer; NOS: not otherwise specified; LLL: left lower lobe; LUL: left upper lobe; RLL: right lower lobe; RML: right middle lobe; RUL: right upper lobe.

**Table 3:** Multivariable competing risk regression for local, nodal, and distant failure outcomes.

	Local Failure			Nodal Failure			Distant Failure		
	aHR	95% CI	*p*	aHR	95% CI	*p*	aHR	95% CI	*p*
Location									
Central	Reference			Reference			Reference		
Peripheral	1.03	0.37–2.83	0.95	1.77	0.63–4.94	0.28	1.60	0.76–3.36	0.22
Age									
<65	Reference			Reference			Reference		
≥65	1.35	0.30–6.16	0.69	0.38	0.13–1.10	0.07	0.56	0.23–1.35	0.20
Gender									
Male	Reference			Reference			Reference		
Female	1.47	0.52–4.18	0.47	0.63	0.24–1.67	0.36	0.75	0.36–1.54	0.43
Race									
Caucasian	Reference			Reference			Reference		
Other	0.64	0.05–7.77	0.73	2.21	0.49–9.94	0.30	1.89	0.59–6.06	0.29
KPS									
<80	Reference			Reference			Reference		
≥80	1.23	0.33–4.58	0.75	2.37	0.71–7.86	0.16	1.75	0.77–3.95	0.18
T stage									
T1	Reference			Reference			Reference		
T2	2.74	0.96–7.79	0.06	1.80	0.65–5.00	0.26	1.26	0.52–3.02	0.61
Histology									
Adenocarcinoma	Reference			Reference			Reference		
Squamous cell carcinoma	0.88	0.29–2.68	0.81	1.60	0.63–4.05	0.33	1.29	0.59–2.80	0.52
NSCLC (NOS)	<0.01	<0.01–<0.01	<0.01	2.13	0.44–10.4	0.35	0.90	0.20–3.97	0.89
Smoking									
Current	Reference			Reference			Reference		
Former	0.63	0.21–1.87	0.40	0.53	0.21–1.36	0.19	0.94	0.42–2.10	0.87
Never	0.64	0.05–7.90	0.73	<0.01	<0.01–<0.01	<0.01	0.71	0.11–4.78	0.73
Tumor location									
LLL	Reference			Reference			Reference		
LUL	1.67	0.26–10.8	0.59	0.38	0.09–1.65	0.20	1.29	0.44–3.76	0.65
RLL	1.10	0.12–9.73	0.93	0.31	0.06–1.72	0.18	0.36	0.09–1.51	0.16
RML	5.01	0.64–39.3	0.13	1.97	0.45–8.60	0.37	1.81	0.38–8.58	0.45
RUL	2.02	0.33–12.3	0.45	0.37	0.09–1.44	0.15	0.69	0.21–2.31	0.54

NSCLC: non-small cell lung cancer; NOS: not otherwise specified; LLL: left lower lobe; LUL: left upper lobe; RLL: right lower lobe; RML: right middle lobe; RUL: right upper lobe.

**Table 4:** Baseline characteristics after propensity score matching.

	Central (*n* = 68)		Peripheral (*n* = 68)		
	N	%	N	%	*p*
Age					1.00
<65	10	14.7	10	14.1	
≥65	58	85.3	58	85.3	
Gender					1.00
Male	36	52.9	36	52.9	
Female	32	47.1	32	47.1	
Race					0.45
Caucasian	61	89.7	57	83.8	
Other	7	10.3	11	16.2	
KPS					0.85
<80	19	27.9	21	30.9	
≥80	49	72.1	47	69.1	
T stage					1.00
T1	52	76.5	52	76.5	
T2	16	23.5	16	23.5	
Histology					0.93
Adenocarcinoma	35	51.5	32	47.1	
Squamous cell carcinoma	28	41.2	31	45.6	
NSCLC (NOS)	5	7.4	5	7.4	
Smoking					0.94
Current	16	23.5	14	20.6	
Former	50	73.5	52	76.5	
Never	2	2.9	2	2.9	
Tumor location					0.94
LLL	11	16.2	10	14.7	
LUL	22	32.4	19	27.9	
RLL	14	20.6	13	19.1	
RML	2	2.9	2	2.9	
RUL	19	27.9	24	35.3	

NSCLC: non-small cell lung cancer; NOS: not otherwise specified; LLL: left lower lobe; LUL: left upper lobe; RLL: right lower lobe; RML: right middle lobe; RUL: right upper lobe.

## Data Availability

Ma and Singh had full access to all the data in the study and take responsibility for the integrity of the data and the accuracy of the data analysis. The data underlying this article cannot be shared publicly for the privacy of the individuals that participated in the study. Research data are stored in an institutional repository and will be shared upon request to the corresponding author.
